# ROR2 has a protective role in melanoma by inhibiting Akt activity, cell-cycle progression, and proliferation

**DOI:** 10.1186/s12929-021-00776-w

**Published:** 2021-11-13

**Authors:** María Victoria Castro, Gastón Alexis Barbero, María Belén Villanueva, Luca Grumolato, Jérémie Nsengimana, Julia Newton-Bishop, Edith Illescas, María Josefina Quezada, Pablo Lopez-Bergami

**Affiliations:** 1 Centro de Estudios Biomédicos, Básicos, Aplicados y Desarrollo (CEBBAD), Universidad Maimónides, 1405 Buenos Aires, Argentina; 2 Consejo Nacional de Investigaciones Científicas y Técnicas (CONICET), 1425 Buenos Aires, Argentina; 3 INSERM U982, Institute for Research and Innovation in Biomedicine, University of Rouen, 76183 Rouen, France; 4 Biostatistics Research Group, Population Health Sciences Institute, Faculty of Medical Sciences, Newcastle University, Newcastle upon Tyne, NE2 4HH UK; 5 Leeds Institute of Medical Research, Leeds, LS2 9JT UK; 6 Centro de Estudios Biomédicos, Biotecnológicos, Ambientales y Diagnóstico, Universidad Maimonides, Hidalgo 775, 6th Floor, Lab 602., 1405 Buenos Aires, Argentina

**Keywords:** ROR2, Akt, Melanoma, Wnt5a, Cell-cycle, Proliferation, Cyclins, Breslow thickness, Survival

## Abstract

**Background:**

Receptor tyrosine kinase-like orphan receptor 2 (ROR2) is a Wnt5a receptor aberrantly expressed in cancer that was shown to either suppress or promote carcinogenesis in different tumor types. Our goal was to study the role of ROR2 in melanoma.

**Methods:**

Gain and loss-of-function strategies were applied to study the biological function of ROR2 in melanoma. Proliferation assays, flow cytometry, and western blotting were used to evaluate cell proliferation and changes in expression levels of cell-cycle and proliferation markers. The role of ROR2 in tumor growth was assessed in xenotransplantation experiments followed by immunohistochemistry analysis of the tumors. The role of ROR2 in melanoma patients was assessed by analysis of clinical data from the Leeds Melanoma Cohort.

**Results:**

Unlike previous findings describing ROR2 as an oncogene in melanoma, we describe that ROR2 prevents tumor growth by inhibiting cell-cycle progression and the proliferation of melanoma cells. The effect of ROR2 is mediated by inhibition of Akt phosphorylation and activity which, in turn, regulates the expression, phosphorylation, and localization of major cell-cycle regulators including cyclins (A, B, D, and E), CDK1, CDK4, RB, p21, and p27. Xenotransplantation experiments demonstrated that ROR2 also reduces proliferation in vivo, resulting in inhibition of tumor growth. In agreement with these findings, a higher ROR2 level favors thin and non-ulcerated primary melanomas with reduced mitotic rate and better prognosis.

**Conclusion:**

We conclude that the expression of ROR2 slows down the growth of primary tumors and contributes to prolonging melanoma survival. Our results demonstrate that ROR2 has a far more complex role than originally described.

**Supplementary Information:**

The online version contains supplementary material available at 10.1186/s12929-021-00776-w.

## Background

Melanoma is the most aggressive form of skin neoplasm causing 80% of skin cancer-related deaths. Furthermore, melanoma incidence continues to increase worldwide and is currently the fifth most common cancer for both men and women in the United States [1]. Melanoma is characterized by the highly prevalent BRAF^V600E^ mutation that renders the MAPK/ERK pathway constitutively active and is critical for melanoma progression. Besides, many other signaling pathways, such as PI3K/Akt, STAT3, Wnt, and Eph/ephrin, are also constitutively activated in melanoma [2, 3]. While targeted and immune-based therapies have improved dramatically the survival rates [4], not all patients benefit, thus establishing the need to identify novel targets that can contribute to improved therapeutic strategies.

Receptor tyrosine kinase-like orphan receptor 1 (ROR1) and 2 (ROR2) are receptors for the Wnt5a ligand that have been proposed as potential high-value targets for cancer therapy [5, 6]. Both receptors play major roles during embryonic development but are strongly downregulated after birth [5]. However, they were shown to express aberrantly in several pathological conditions in the adult. Interestingly, ROR2 possesses dual roles in cancer and can act to either suppress or promote carcinogenesis in different cancers [7]. The up-regulation of ROR2 have been described in osteosarcoma [8], squamous cell carcinoma [9], non-small cell lung carcinoma [10], pancreatic ductal adenocarcinoma [11], renal cell carcinoma [12], papillary thyroid carcinoma [13], leukemia [14], gastrointestinal cancer [15], and cancers from the prostate [16], head and neck [17], ovarian [18, 19], breast [20], and cervix [21]. In most of them, high levels of ROR2 correlated with increased tumorigenic properties, such as proliferation, migration, invasion, anchorage-independent growth, epithelial-to-mesenchymal transition (EMT), and in vivo tumor development. On the other hand, ROR2 has a tumor-suppressive function in endometrial [22] and colon cancer [23] as well as in medulloblastoma [24] and hepatocellular [25], and gastric carcinoma [26]. These studies revealed that ROR2 over-expression inhibited proliferation, migration, invasion, colony formation, cell-cycle progression, and tumor formation in vivo, together with an increase in apoptosis and in vitro sensitivity to chemotherapy. In both scenarios, there is very limited information regarding the mechanisms by which ROR2 regulates these processes in cancer.

ROR2 has been scarcely investigated in melanoma. It was shown that murine Ror2 contributed to migration and metastasis [27] and that ROR2 expression associates with a more invasive phenotype [28] and is necessary for the Wnt5A-mediated metastasis of melanoma cells [29]. However, the implicated mechanisms have not been investigated. To elucidate the function of ROR2 in melanoma in greater depth, the present work studied the role of ROR2 in the regulation of cell proliferation and cell-cycle progression, as well as the underlying molecular mechanisms.

## Methods

### Cell culture

Melanoma lines were provided by Dr. Zeev Ronai (Sanford Burnham Prebys Medical Discovery Institute) except for the M2 cell line that was provided by Sergio Alvarez (IMIBIO-CONICET) [30]. Cells were maintained in DMEM supplemented with 10% fetal bovine serum (FBS, Gibco), 100 U/ml penicillin and 100 mg/ml streptomycin (Invitrogen), at 37 °C and 5% CO_2_. The cell lines were free of mycoplasma contamination and were authenticated by short tandem repeat analysis as described [31]. Stimulation with Wnt5a was performed as described [32]. Synchronization of cell cultures were obtained by incubation for 80 h in serum-free DMEM. The concentration of LY294002 (154447-36-6, Calbiochem) was 20 µM. DMSO was used as control.

### shRNA constructs, over-expression system and viral infection

The shRNAs against ROR2 were described previously [33]. The targeting sequences are C2 shRNA 5ʹ-CTGGGTGTATGCCCTCATGAT-3ʹ and C4 shRNA 5ʹ-CCCTGGTGCTTTACGCAGAAT-3ʹ. Human ROR2 was cloned into the VIRSP vector. Generation of viral particles and stable transduction were performed as described [32, 34].

### Proliferation assays

The experiments were performed as described [35]. The doubling time (DT) was calculated as

$${\text{DT}} = {\text{ln}}\left( {2} \right) \times {\text{96 h}}/{\text{ln}}\left( {{\text{number of cells at 96 h}}/{\text{number of cells at }}0\;{\text{h}}} \right).$$

### Western blotting

Cell lysates were collected and processed as described [34]. The antibodies used are described in Additional file 1: Table S1. The band intensities in the phosphoprotein blots were normalized with those of the total proteins obtained from the same blots after stripping and reprobing. To draw a conclusion in a particular experiment at least three biological (independent) replicates of paired samples were examined to calculate the mean and standard deviation. The log transformation of FC values was calculated to obtain a more symmetric distribution that better suits the normality assumptions of the subsequent t-test. Tumor tissue from xenotransplanted mice were minced from the surrounding skin, homogenized and embedded in RIPA Buffer supplemented with protease and phosphatase inhibitors in a relation of 350 µl Buffer/20 mg tissue. The mixture was incubated for 30 min in agitation at 4 °C. Finally, it was centrifuged at 10,000 rpm, at 4 °C for 10 min, and supernatant was recovered and stored at − 20 °C.

### Cell-cycle analysis

Cells were collected, washed in PBS, fixed in 4% PFA and permeabilized (PBS, 1% Saponin, 10 mM HEPES). The cells were washed in Wash Buffer (PBS, 0.5% Saponin, 10 mM HEPES), and pelleted at 3000 rpm for 5 min. The cells were resuspended in 500 µl of Staining Buffer [PBS, 1% Saponin, 10 mM HEPES, 50 µg/µl propidium iodide (PI; Cayman Chemicals, 14289) and RNAse 100 µg/µl] and incubated at 4 °C for 20 min in the dark. DNA content analyses were conducted using a FACS Canto II flow cytometer (BD Biosciences) and analyzed using FlowJo software.

### In vivo assays

N:NIH(S)-nu nude mice were injected subcutaneously with 2 × 10^6^ A375-Empty or -ROR2 cells and tumor growth was monitored twice a week. Tumor sizes were measured using a caliber, and tumor volumes were calculated using the formula: volume = (2 × width × length)/2. Tumors were harvested, paraffin embedded and sectioned.

### Immunohistochemistry

Immunohistochemistry staining of tissue samples was performed as described [35]. Antigen retrieval was by heat-induced epitope retrieval using Citrate Buffer (10 mM, pH 6) for ROR2, and Ki67 or Tris-EDTA Buffer (Tris 10 mM and EDTA 1 mM, pH 9) for p-Akt-T308 at 100 °C for 20 min in microwave. Primary antibodies were incubated ON at RT in a humidified chamber. The slides were analyzed using an optic microscope (BX40, Olympus Optical Corporation, Tokyo, Japan) and imaged using a coupled digital camara (390CU 3.2 Mega Pixel CCD Camera, Micrometrics, Spain).

### Immunofluorescence

Cells were seeded in slides and 48 h later were fixed in DMEM with 4% PFA for 10 min at RT. Slides were washed in PBS and placed in permeabilization solution (0.5% Triton-X 100, 3 mM Cl2Mg, 6.84% Sucrose) on ice for 5 min. Then, slides were blocked in PBS 3% BSA during 1 h at RT. Next, the slides were incubated with RB (sc-102) antibody, overnight at 4 °C in a humidified chamber. After the incubation, three washes with PBS were made, and slides were incubated with secondary antibody (Alexa Fluor 488) for 1 h in the dark at RT. Finally, slides were mounted with Vectashield DAPI ANTIFADE Mounting Medium (Vector). Pictures were taken on Nikon C1 Plus Microscopy and analyzed on FIJI. We calculated a CTCF (Corrected Total CellSection Fluorescence) coefficient as described [32].

### Flow cytometry

Single-cell suspensions were fixed (4% PFA) and permeabilized (PBS, 1% Saponin, 10% FBS). Then the cells were washed with PBS and blocked with purified anti-mouse serum diluted in FACS buffer (PBS, 2% Serum). After that, the cells were incubated with primary antibodies or respective isotype controls (preimmune rabbit sera or isotype matched mouse Ig) for 30 min at 4 °C. Unconjugated antibodies to ROR2 (34045) from QED Bioscience Inc., and Cyclin E1 (sc-481), from Santa Cruz Biotechnologies were used. The corresponding secondary antibodies (Alexa Fluor 488) were added for 30 min at RT in dark. In the case of ROR2, surface staining before permeabilization was made in an independent experiment. Cells were acquired on a FACS Canto II flow cytometer (BD Biosciences) and analyzed using FlowJo software. Both Forward Scatter and Side Scatter were used to identify the cell populations and to determine changes in size or granularity of the cells. The protein levels were quantified as the mean fluorescence intensity (MFI) after subtraction of control Ab MFI.

To determine apoptotic cell death the cells were seeded on a 60-mm dish at a density of 3 × 10^5^ cells. The following day they were exposed to 20 µM LY294002 (or DMSO as a control) for 48 h. Cells were washed twice with PBS and resuspended in 100 µl of Annexin V binding buffer (pH 7.4) (10 mM HEPES, 140 mM NaCl, 2.5 mM CaCl_2_). Then, annexin V-Alexa Fluor 488 (BD Biosciences) was added and incubated for 15 min under dark conditions. Propidium iodide (0.1 µg/ml; PI; Cayman chemicals, 14289) was added just prior to signal acquisition. Cells were analyzed using a FACS Canto II flow cytometer (BD Biosciences) and analyzed with FlowJo software.

### Resazurin reduction assay

To evaluate changes in cellular viability we used the colorimetric rezasurin reduction assay. For this, 7500 cells/well were plated in a 96-well plate. To evaluate the effect of PI3K/Akt pathway on viability, were incubated with DMEM (red phenol free) 10% FBS supplemented with increasing concentrations of LY294002 (154447-36-6, Calbiochem) for 48 h. After this, the rezasurin solution was added to each well in a final concentration of 0.4 mM and incubated for 4 h. The absorbance (Optical Density (OD)) was detected at 570 and 600 nm with a μQuant microplate reader (Biotek Instrument). The percentage of viability was calculated as described [31].

### Bioinformatic analysis

*ROR2* expression was tested for association with tumor AJCC T stage, mitotic rate, ulceration and melanoma-specific survival (MSS) in the Leeds Melanoma Cohort (LMC), a large population-based cohort with transcriptomes from 703 formalin-fixed paraffin embedded (FFPE) samples from primary melanoma patients with a median follow-up time of 8 years (Accession number EGAS00001002922, National ethical approval MREC 1/03/57 and PIAG3-09(d)/2003) [36]. *ROR2* was also tested for association with a gene expression signature (LMC classes) that we earlier reported as prognostic in primary melanoma and indicative of the EMT pathway activation [37]. STATA 14 (StataCorp, Texas, USA) was used to conduct the Mann–Whitney test for group comparisons as well as Cox proportional hazard regression and Kaplan–Meier curves for survival analysis after expression dichotomization by median split. Dataset GSE57715 (n = 297) was used to determine the relationship between ROR2 expression and Breslow thickness.

### Statistics

All experiments were performed at least three times. A mean and standard error was derived from all repeated experiments. Student’s t-test was performed to compare treated groups to vehicle control. Values of p < 0.05 were considered statistically significant. Statistical analyses were conducted using software from Graph-Pad Prism. The number of independent experiments and specific statistical analyses used in each experiment are indicated in the figure legends.

## Results

### ROR2 inhibits melanoma cell proliferation

**Fig. 1:**
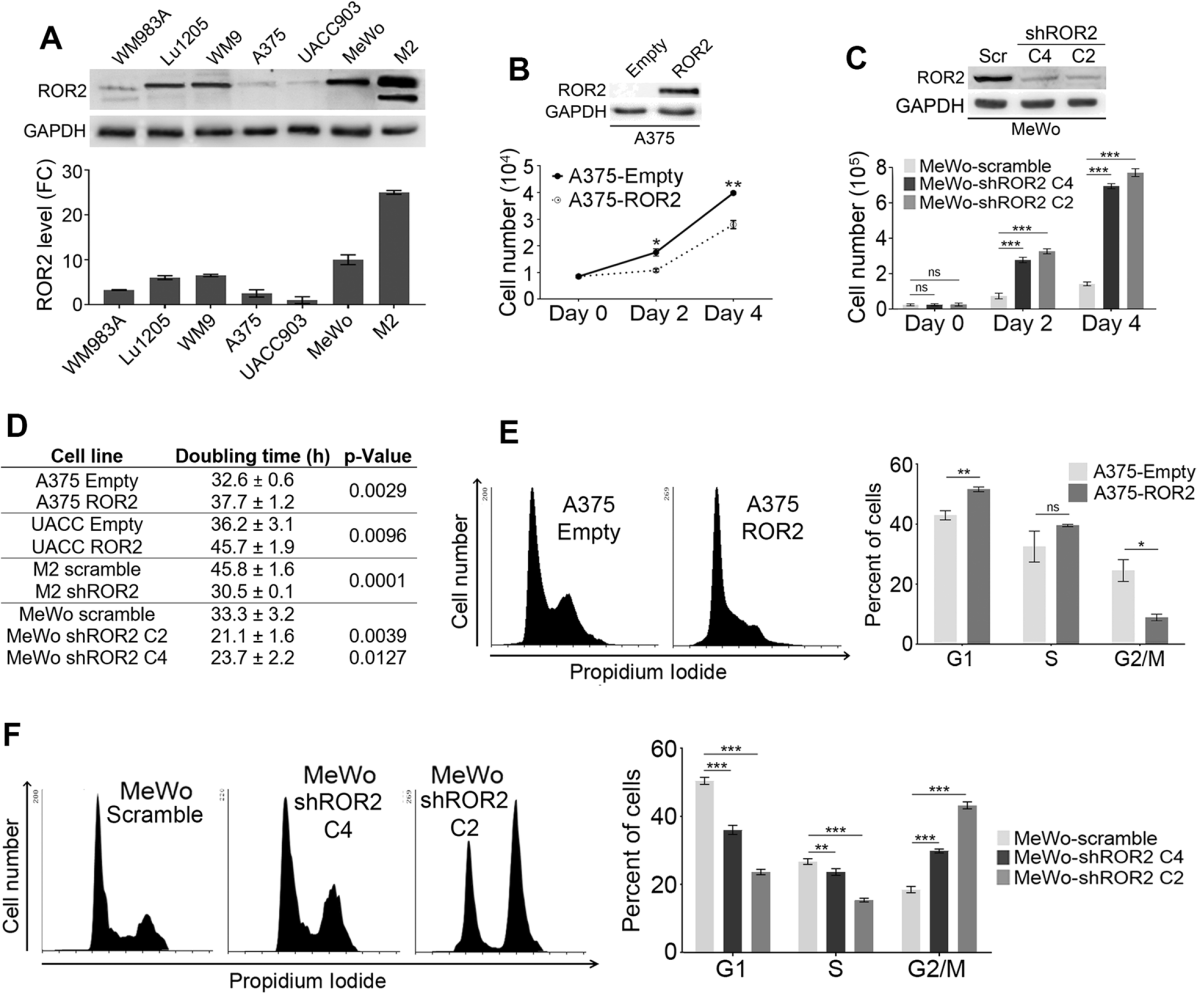
ROR2 inhibits the proliferation of melanoma cells. **A** Expression of ROR2 in human melanoma cell lines. ROR2 levels were normalized to GAPDH and expressed as fold change (FC) relative to UACC903 cells. **B**, **C** ROR2 inhibits proliferation of melanoma cells. Crystal violet assays were performed in A375 cells stably transduced with either control (Empty) or a ROR2-expressing plasmid (**B**) and in MeWo cells stably transduced with either control (scramble) plasmid or two shRNA for ROR2 (C4 and C2) (**C**). Statistical significance was tested by a one-tailed Student’s T-Test (**B**) or ANOVA (**C**). ROR2 overexpression and silencing was determined by western blot. **D** ROR2 expression alters the doubling time of melanoma cells. **E**, **F** ROR2 impairs cell-cycle progression. Flow cytometry analysis of PI-stained A375 cells overexpressing ROR2 (**E**) and MeWo cells upon silencing of ROR2 (**F**). Bar graph shows the mean ± S.D. of the percentage of cells in each phase of the cell-cycle. Statistical significance was tested by a one-tailed Student’s T-Test (**E**) or ANOVA (**F**). *: p < 0.01, **: p < 0.001, ***: p < 0.0001, n.s.: not significant

To investigate the role of ROR2 in melanoma cell proliferation we used both gain- and loss-of-function approaches. Based on western blot analysis of a panel of melanoma cell lines (Fig. 1A), cell lines A375 and UACC903, expressing low levels of ROR2, were selected for gain-of-function studies and stably transduced with an expression vector encoding human ROR2 or Empty vector as a control (Fig. 1B and Additional file 2: Fig. S1A, B). Flow cytometry analysis of both permeabilized and intact cells revealed that most of the exogenous ROR2 protein localizes, as expected, at the plasma membrane (Additional file 2: Fig. S1B, C). Importantly, exogenous ROR2 levels were within the physiological range, as indicated by comparison with endogenous ROR2 levels in HeLa cells (Additional file 2: Fig. S1D). Overexpression of ROR2 in both A375 and UACC903 cells significantly reduced cell proliferation compared to control cells (Fig. 1B and Additional file 2: Fig. S1A). To silence ROR2 expression, cell lines M2 and MeWo, displaying high ROR2 levels, were stably transduced with retroviral particles encoding two short-hairpin RNAs (shRNAs) specific for ROR2 (C4 and C2) or a scramble sequence (control shRNA) (Fig. 1C and Additional file 2: Fig. S1E, F). The cells transduced with ROR2 shRNA (M2-shROR2 and MeWo-shROR2) showed a significant increase in proliferation compared to the respective scramble cells (Fig. 1C and Additional file 2: Fig. S1E). In addition, manipulation of ROR2 levels significantly altered the doubling time of the four cell lines analyzed (Fig. 1D). These results demonstrate that ROR2 inhibits melanoma cell proliferation.

We next analyzed whether ROR2 expression affects the cell-cycle by measuring the DNA content of asynchronous cells. The overexpression of ROR2 induced a significant decrease in G2/M cells with a concomitant increase of cells in G1 (A375) or S phase (UACC903) (Fig. 1E and Additional file 2: Fig. S1G). The opposite results were observed in both M2 and MeWo upon ROR2 silencing. Knockdown of ROR2 in MeWo cells significantly decreases the number of cells in both G1 and S phases while it increases the number of cells in G2/M (Fig. 1F). Silencing of ROR2 in M2 cells also altered the cell-cycle, most noticeably by increasing the cells at the G2/M phase (Additional file 2: Fig. S1H). Altogether, these results indicate that ROR2 inhibits proliferation by affecting cell-cycle progression.

### ROR2 slowed down cell-cycle progression by regulating the levels of major proteins implicated in cell-cycle control

**Fig. 2:**
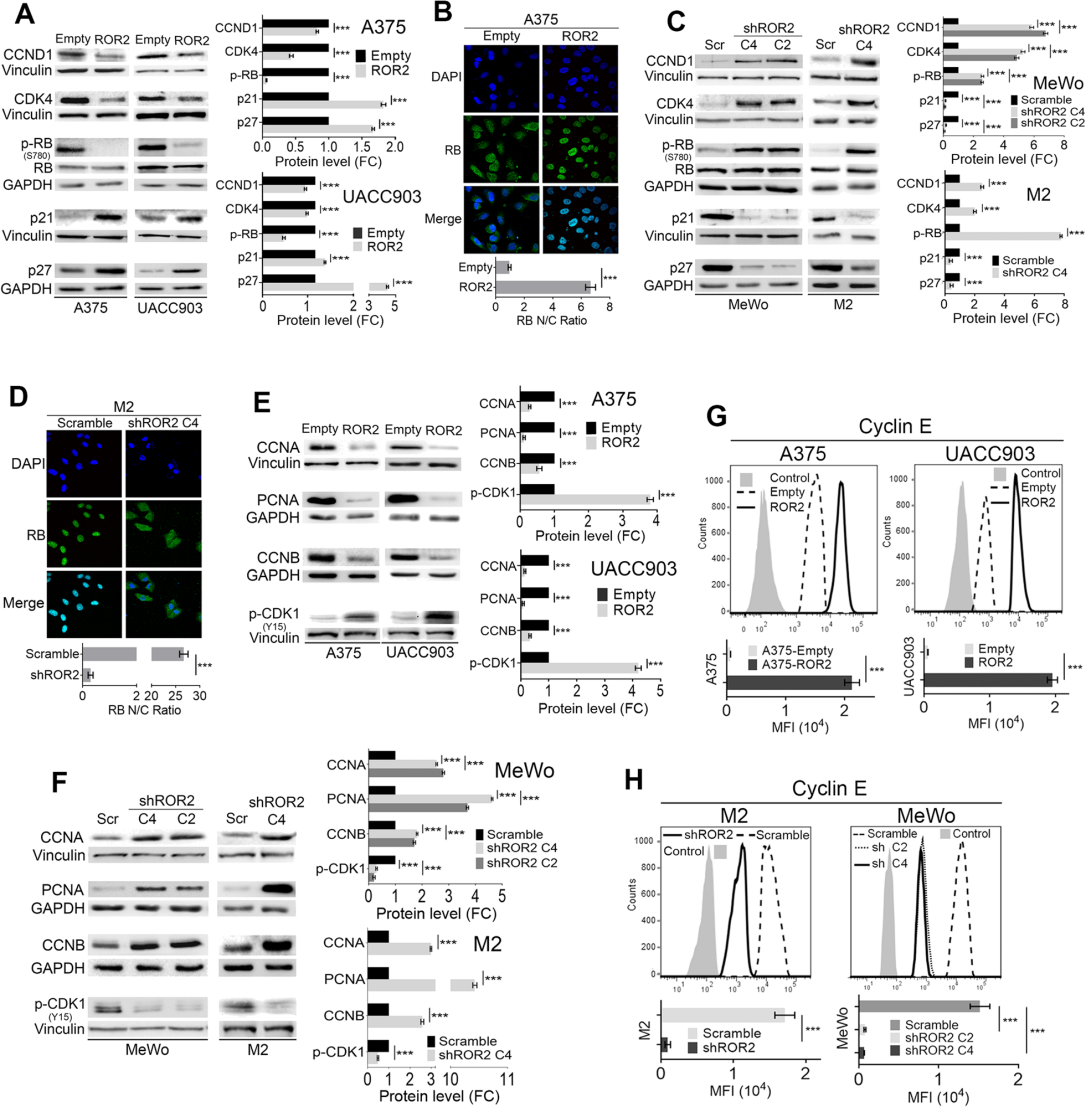
ROR2 regulates the expression of cell-cycle markers and delays cell-cycle progression. Western blot analysis of the expression of cell-cycle markers in A375 and UACC903 overexpressing ROR2 (**A**, **E**) or M2 and MeWo upon ROR2 silencing (**C**, **F**). The graphs show the mean ± S.D. of each protein’s levels in cells transduced with either ROR2 cDNA or shRNA normalized to the corresponding loading control and expressed as the fold change (FC) relative to the corresponding control cell line (Empty or scramble). Statistical significance was tested by a one-tailed Student’s T-Test or ANOVA (MeWo) (n = 3). **B**, **D** Assessment of RB localization by immunofluorescence upon ROR2 overexpression (**B**) or silencing (**D**). A375-Empty and A375-ROR2 (**B**) or M2-scramble and M2-shROR2 (**D**) were stained with DAPI and anti-RB antibody. Graphs show the quantification of RB nuclear/cytosolic localization. Statistical significance was tested by a one-tailed Student’s T-Test (n = 3). **G**, **H** Flow cytometry analysis of Cyclin E expression upon ROR2 overexpression (**G**) or silencing (**H**). Bar graph shows the mean of Cyclin E MFI (Mean Fluorescence Intensity) ± S.D. Statistical significance was tested by a one-tailed Student’s T-Test (**G**) or ANOVA (**H**, MeWo) (n = 3). *: p < 0.01, **: p < 0.001, ***: p < 0.0001, n = 3

To further study the role of ROR2 on cell-cycle progression we assessed the expression levels of protein markers of each cell-cycle phase. We determined that ROR2 overexpression in both A375 and UACC903 cells significantly decreased the expression of Cyclin D1 (CCND1) and CDK4, two key G1 phase regulators (Fig. 2A). As expected, phosphorylation of the RB1 tumor suppressor protein was dramatically reduced by ROR2 overexpression (Fig. 2A) along with a rise in RB nuclear localization (Fig. 2B). Along these lines, ROR2 increased the levels of the CDK inhibitors p21 and p27 (Fig. 2A). These results were confirmed by ROR2 knockdown (Fig. 2C, D). Moreover, we determined that ROR2 overexpression in both A375 and UACC903 cells significantly decreased the expression of the late cell-cycle markers PCNA and Cyclin A (CCNA) (Fig. 2E). In contrast, ROR2 silencing increased the levels of both proteins (Fig. 2F). ROR2 also decreased the activity of the CDK1/Cyclin B1 complex critically implicated in mitosis entry as evidenced by the reduced level of Cyclin B (CCNB) and the increased inhibitory phosphorylation of CDK1 at Y15 (Fig. 2E). The opposite results were observed upon ROR2 knockdown (Fig. 2F). Another protein importantly implicated in cell-cycle control is Cyclin E that peaks at the beginning of the S phase and needs to be degraded for the cell-cycle to progress. Flow cytometry analysis demonstrated that Cyclin E expression increased in both A375 and UACC903 cells overexpressing ROR2 (Fig. 2G) and decreased upon ROR2 silencing in M2 and MeWo cells (Fig. 2H). These results show that ROR2 expression has a negative impact on proteins implicated in both G1 to S and S to G2/M transitions resulting in fewer cells reaching the G2/M phase and a decreased proliferation rate.

**Fig. 3:**
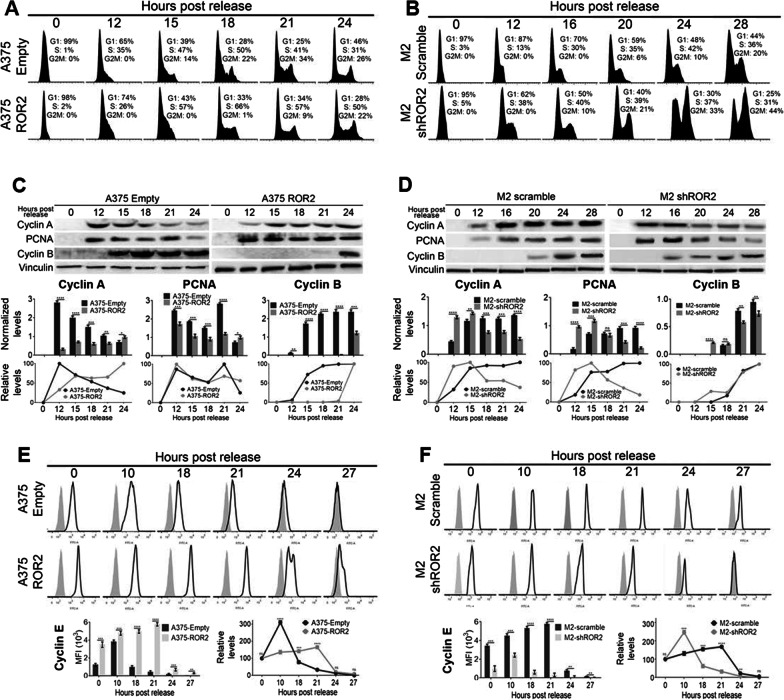
ROR2 impairs cell-cycle progression in synchronized cells. **A**, **B** Flow cytometry analysis of PI-stained A375 overexpressing ROR2 (**A**) and M2 cells (**B**) upon silencing of ROR2. Cells were synchronized and then released by addition of 10% serum as described in “Methods”. The analysis was performed as described in Fig. 1E, F. **C**, **D** Effect of ROR2 on the expression of cell-cycle markers. Protein extracts from A375 cells overexpressing ROR2 (**C**) and M2 cells upon silencing of ROR2 (**D**) treated as described above were analyzed by western blot with the indicated antibodies. Vinculin was used as a loading control. The upper graphs show the mean ± S.D. of the protein level of each protein at each time-point normalized to the respective loading control. In the lower graphs (line charts), the time-point with the maximal expression was assigned a value of 100 and the relative expression level of the remaining time points were calculated accordingly. The blots displayed are representative of three independent experiments. Statistical significance was tested by one-tailed Student’s T-Test, n = 3. **E**, **F** Flow cytometry analysis of Cyclin E expression in A375 overexpressing ROR2 (**E**) and in M2 (**F**) upon silencing of ROR2 cells that were treated as described above. Light-grey filled histogram corresponds to control (isotype) antibody and open histograms to Cyclin E antibody. The upper graphs show the mean ± S.D. of Cyclin E MFI at each time-point (from three independent experiments). In the line chart the level of Cyclin E was plotted relative to the time point with the maximal level. Statistical significance was tested by a one-tailed Student’s T-Test. *: p < 0.01, **: p < 0.001, ***: p < 0.0001, n = 3. n.s.: not significant

To gain further insight into ROR2’s role in this process, we synchronized cells in the G1 phase by serum starvation. Upon release from the induced G1 block by the addition of serum, cells were harvested at different time-points and both the cell-cycle distribution and the abundance of cell-cycle-related proteins were determined. Expression of ROR2 slowed down both the G1 to S (12 h time-point) and the S to G2/M transitions (15 h to 21 h time-points) (Fig. 3A), resulting in an overall 6 h delay in cell-cycle progression (Fig. 3A, compare A375-Empty (18 h) with A375-ROR2 (24 h)). Similar results but in the opposite direction were observed upon ROR2 silencing. In this case, ROR2 silencing accelerates cell-cycle progression by approximately 8 h (compare 12 and 20 h time-point for changes in S phase and 20 and 28 h time-points for changes in G2/M, Fig. 3B). These alterations are in agreement with the differences in doubling time described in Fig. 1D. The inhibitory effect of ROR2 on the cell-cycle was also observed by the different patterns of re-expression and degradation of proteins from the Cyclin family and PCNA upon serum restoration. Cyclin A levels, a late S-phase marker, peaked at 12 h after G1 release and then progressively decreased in A375-Empty cells (Fig. 3C). In contrast, in A375-ROR2 cells, Cyclin A levels at 12 h were low and increased slowly until peaking at 24 h (Fig. 3C). The slow build-up of Cyclin A levels induced by exogenous ROR2 was also observed in M2-scramble cells that, as shown above, express high levels of endogenous ROR2. Silencing ROR2 in these cells (M2-shROR2 cells) allows for Cyclin A degradation from 16 h post-release onward (Fig. 3D). These results suggest that both Cyclin A expression and degradation are affected by ROR2. Similar changes were observed in PCNA levels (Fig. 3D). Likewise, ROR2 expression markedly delayed Cyclin B re-expression (required for mitosis entry) until 24 h compared with the earlier expression (15–18 h) observed in A375-Empty cells (Fig. 3C). Meanwhile, after peaking at 10 h, Cyclin E is rapidly degraded in A375-Empty cells (Fig. 3E). In contrast, A375-ROR2 cells failed to timely degrade Cyclin E as evidenced by the gradual increase in its levels until 21 h (Fig. 3E). A similar effect of ROR2 in Cyclin E level was observed in experiments of ROR2 knockdown (Fig. 3F). Altogether, these results demonstrate that ROR2 strongly regulates the level of expression of proteins regulating cell-cycle progression which explains the observed effects of ROR2 in the cell-cycle distribution and proliferation of melanoma cells.

### The negative effect of ROR2 in cell proliferation is mediated by Akt inhibition

**Fig. 4:**
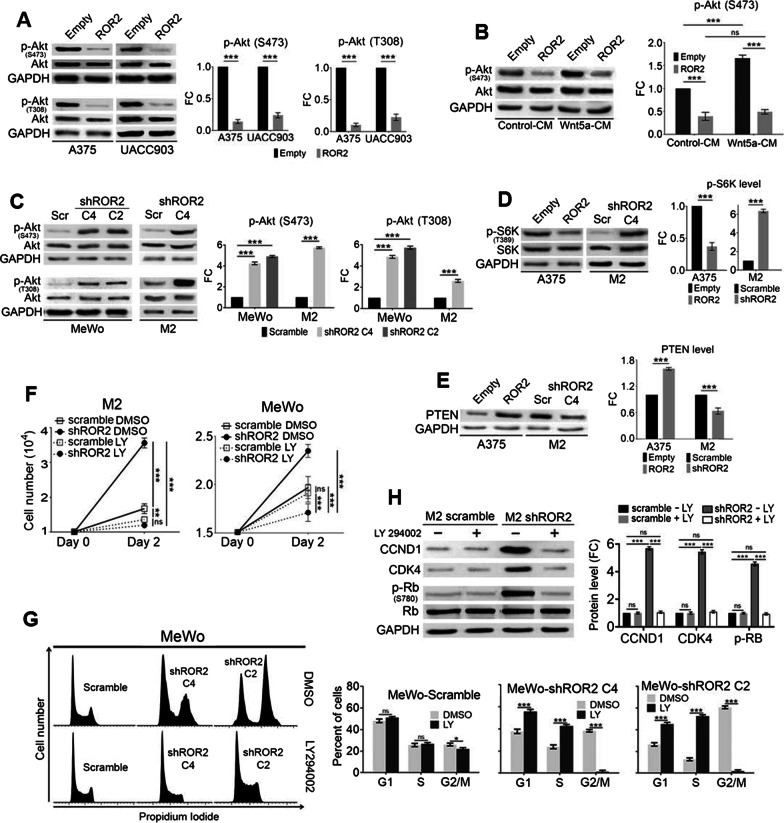
The effect of ROR2 in proliferation is mediated by inhibition of Akt phosphorylation. **A** Expression of ROR2 in both A375 and UACC903 cells inhibits Akt-T308 and -S473 phosphorylation. The graphs show the mean ± S.D. of p-Akt levels normalized to total Akt and expressed as the fold change (FC) relative to the corresponding control cell line (empty or scramble). Statistical significance was tested by a one-tailed Student’s T-Test (n = 3). **B** ROR2 expression in A375 prevents Akt phosphorylation by Wnt5a. The cells were incubated with Wnt5a conditioned media for 30 min and Akt-S473 phosphorylation was assessed as in A. Statistical significance was tested by ANOVA (n = 3). **C** Silencing of ROR2 increased Akt phosphorylation in M2 and MeWo cells. Akt phosphorylation upon ROR2 silencing in M2 and MeWo cells was assessed as in **A**. Statistical significance was tested by a one-tailed Student’s T-Test (M2) or ANOVA (MeWo) (n = 3). **D** ROR2 regulates phosphorylation of S6K. The graphs show the mean ± S.D. of the p-S6K levels normalized to total S6K and expressed as the fold change (FC) relative to the corresponding control cell line (Empty or scramble). Statistical significance was tested by a one-tailed Student’s T-Test (n = 3). **E** ROR2 increases PTEN expression. Western blot analysis of PTEN expression in A375-Empty, A375-ROR2, M2-scramble and M2-shROR2 cells. Bar graph shows the mean ± S.D. (from three independent experiments) of PTEN levels normalized to the loading control (GAPDH) and expressed as the fold change (FC) relative to control cells. The blots displayed are representative of three independent experiments. Statistical significance was tested by one-tailed Student’s T-Test, n = 3. **F** LY294002 (LY) inhibited the effects of ROR2 in cell proliferation. The cells were grown for two days in the presence or absence of 20 μM LY294002. Graphs show the mean ± S.D. of the number of cells. Statistical significance was tested by a one-tailed Student’s T-Test (n = 3). **G** LY294002 prevented the accumulation of cells in G2/M in M2-shROR2 cells. M2-scramble and M2-shROR2 cells were incubated with 20 μM LY294002 for 48 h and analyzed by flow cytometry. Bar graph shows the mean ± S.D. of the percentage of cells in each phase of the cell-cycle. Statistical significance was tested by a one-tailed Student’s T-Test. **H** LY294002 counteracts the increase in cell-cycle markers induced by ROR2 silencing. The cells were treated with 20 μM LY for 12 h. The graphs show the mean ± S.D. of each protein’s levels in cells transduced with ROR2 shRNA normalized to the corresponding loading control and expressed as the fold change (FC) relative to scramble cells. Statistical significance was tested by ANOVA (n = 3). *: p < 0.01, **: p < 0.001, ***: p < 0.0001, n = 3. n.s.: no significant

To elucidate the molecular pathways responsible for the anti-proliferative role of ROR2 we first evaluated the effects of ROR2 on the PI3K/Akt pathway, a signaling cascade that is usually constitutively active in melanoma. Overexpression of ROR2 abrogated phosphorylation of both Akt-T308 and -S743, two critical residues for Akt activity, in both A375 and UACC903 cells (Fig. 4A). Moreover, ROR2 overexpression in A375 cells also prevents phosphorylation of Akt induced by Wnt5a, the canonical ROR2 ligand (Fig. 4B). Despite M2 and MeWo cells also present constitutive Akt activation, ROR2 silencing further increased Akt phosphorylation at both residues (Fig. 4C). Along this line, gain- and loss-of-function experiments showed that ROR2 inhibited the phosphorylation of S6K, an Akt target (Fig. 4D), confirming that ROR2 inhibits PI3K/Akt pathway activity. We also determined that ROR2 regulates PTEN levels in both A375 and M2 cells (Fig. 4E) providing a plausible mechanisms for the regulation of Akt phosphorylation by ROR2. However, other mechanisms contribute to this process since ROR2 still regulates Akt phosphorylation in UACC903 cells that are PTEN-null [38].

To evaluate the biological relevance of the increased Akt activity induced by ROR2 silencing we used the PI3K/Akt pathway inhibitor LY294002 (LY). Our goal was to use the inhibitor to counteract the effect of ROR2 silencing on Akt activation. Although the p-Akt level is much higher in ROR2-knockdown cells, we corroborated it was similarly inhibited by LY in both M2-scramble and M2-shROR2 cells (Additional file 2: Fig. S2A). Whereas LY had a small effect on both M2-scramble and MeWo-scramble proliferation, it potently inhibited (66% and 54% inhibition in M2 and MeWo, respectively) the proliferation of both M2 and MeWo cells transduced with ROR2 shRNA (Fig. 4F). This effect on proliferation was also manifested as a decrease in the viability of M2-shROR2 cells after a 48 h incubation with LY. In dose–response experiments, the IC25 for LY was 65.3 μM for M2-scramble cells and 16.3 μM for M2-shROR2 cells (Additional file 2: Fig. S2B). These changes were not due to the induction of apoptosis by LY since cell death was negligible in both cell lines (Additional file 2: Fig. S2C). Further, incubation with LY completely prevented the accumulation of G2/M cells observed upon ROR2 silencing (Fig. 4G). To determine the exact mechanisms by which regulation of Akt activity by ROR2 regulates proliferation we measured the expression of proteins implicated in cell-cycle control upon simultaneous LY treatment and ROR2 knockdown. Incubation with LY counteracted the increase in CCND1, CDK4, and p-RB observed upon ROR2 silencing (Fig. 4H) indicating that the effects of ROR2 on cell growth were mediated by its negative effects on Akt activity. These results also reveal that ROR2 silencing generated a highly proliferative phenotype that is extremely dependent on the PI3K/Akt pathway. Together with our previous results, these data indicate that the negative effect of ROR2 in cell proliferation is mediated by the inhibition of Akt phosphorylation and activity, which impacts the expression of proteins implicated in cell-cycle progression.

### ROR2 inhibits in vivo tumor growth

**Fig. 5:**
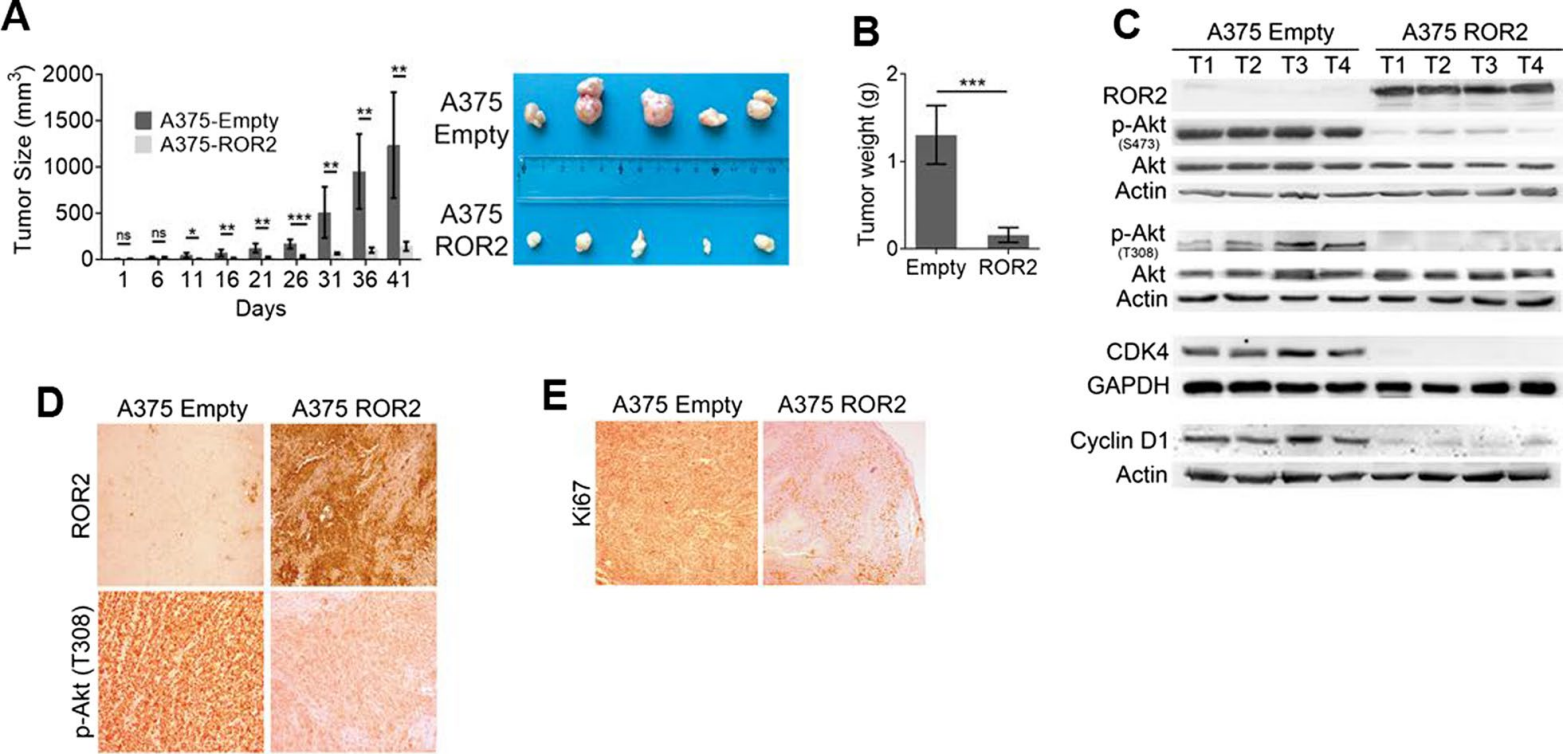
ROR2 inhibits tumor growth in vivo. **A** ROR2 expression inhibits tumor growth. A375-Empty and A375-ROR2 cells were xenotransplanted in nude mice (6 per group) and tumor volume was monitored twice weekly and presented as a mean ± S.D. Photographs of dissected tumors are shown. Statistical significance was tested by a one-tailed Student’s T-Test (n = 3). *: p < 0.01, **: p < 0.001, ***: p < 0.0001, n = 3. n.s.: no significant. **B** Tumors from mice injected with A375-ROR2 cells presented a reduced weight. The bar graph shows the mean ± S.D of the tumor weight from mice injected with A375-Empty and A375-ROR2. Statistical significance was tested by a one-tailed Student’s T-Test. ***: p < 0.0001. **C** Tumors from A375-ROR2 mice present reduced levels of p-Akt and of proliferation markers. Western blot analysis in tumors derived from xenografted A375-Empty and A375-ROR2 cells. T1 to T4 represent four different tumors in each group. GAPDH and β-actin were used as loading controls. **D** Tumors expressing ROR2 lack Akt phosphorylation. Immunohistochemistry (IHC) staining was used to examine both ROR2 and p-Akt levels in paraffin-stained tumor sections. Representative images at 40× magnification are shown. **E** ROR2 inhibits cell proliferation in vivo. Representative IHC staining of proliferative nuclei with Ki67 in xenograft tumor sections (40× magnification)

We next performed xenotransplantation experiments to evaluate the role of ROR2 on in vivo melanoma growth. By the 2nd week after injection, the tumors generated by A375-Empty cells were already significantly larger than those in mice injected with A375-ROR2 cells (Fig. 5A). At the end of the experiment, the A375-ROR2 tumors were more than ten times smaller tumors than A375-Empty tumors (Fig. 5A) with a remarkable decrease in tumor weight (Fig. 5B). In agreement with in vitro data, tumors from mice injected with A375-ROR2 showed a decreased expression of Cyclin D1 and CDK4 along with a marked decrease in both Akt-T308 and -S473 phosphorylation compared with control tumors (Fig. 5C, D). Along this line, the A375-Empty tumors proliferate more than those derived from A375-ROR2 cells as indicated by Ki67 staining (Fig. 5E). These results demonstrate that ROR2 inhibits in vivo tumor growth through decreased proliferation.

### ROR2 has a protective role in primary melanoma

**Fig. 6:**
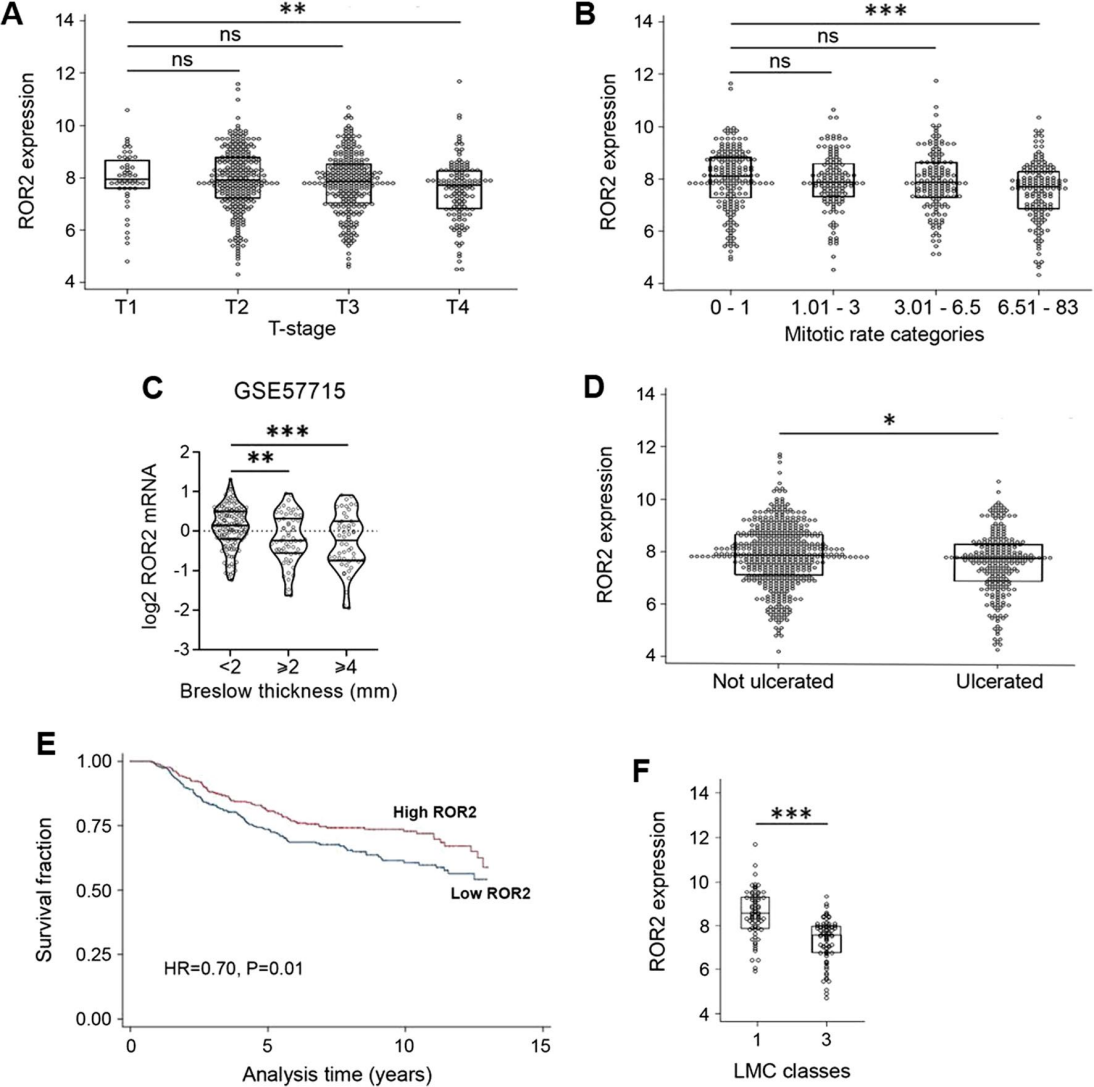
ROR2 has a protective role in primary melanoma. ROR2 expression in the Leeds Melanoma Cohort (n = 703) correlates negatively with the AJCC T-stage (**A**) and mitotic rate (**B**). **C** ROR2 expression negatively correlates with Breslow thickness. Violin plot of *ROR2* mRNA levels in dataset GSE57715. ROR2 levels were compared in patients with Breslow depth lower than 2 mm (n = 135), between 2 and 4 mm (n = 59), and greater than 4 mm (n = 50). Statistical significance was tested by ANOVA. **D** ROR2 expression negatively correlates with ulceration in the LMC. **E** High ROR2 expression correlates with longer melanoma specific survival (MSS). Kaplan–Meier MSS analysis of 703 primary melanomas stratified by ROR2 median split. **F** *ROR2* mRNA expression is higher in LMC class 1 with the best melanoma specific survival. The LMC classes with the best (class 1) and worst (class 3) survival were compared for *ROR2* mRNA levels [37]. Mann–Whitney test for group comparisons and Cox proportional hazards regression for survival analysis. n.s.: no significant, *: p < 0.01, **: p < 0.001, ***: p < 0.0001

Since the results above show that ROR2 inhibits melanoma cell proliferation and tumor growth, we wanted to determine whether ROR2 expression in human melanomas associates with tumor features related to proliferation. Using whole transcriptome data derived from 703 primary melanomas from the Leeds Melanoma Cohort (LMC) [36], we found that ROR2 level is highest in tumors at the T1 stage and significantly higher than tumors at the T4 stage (Fig. 6A). Similarly, ROR2 expression is highest in tumors with reduced mitotic rate and decreased in tumors with high mitotic rate (i.e. greater than 6.5) (Fig. 6B). We further investigated this issue in another large cohort composed of 244 primary melanomas (GSE57715). Since T-stage was not reported for this dataset, ROR2 expression was compared with Breslow’s depth. Again, ROR2 level was higher in thin tumors (less than 2 mm) than in tumors with intermediate thickness (between 2 and 4 mm). ROR2 level was further reduced in thicker tumors (greater than 4 mm) (Fig. 6C). These results indicate that high ROR2 levels correlate with thin tumors and reduced proliferation.

In line with the findings above, tumors with lower ROR2 levels were more likely to be ulcerated, a predictor of poor outcome (Fig. 6D). In agreement with this observation, higher ROR2 expression correlated with longer melanoma-specific survival (Fig. 6E). We previously reported a prognostic gene expression signature (LMC classes) in the LMC [37]. Interestingly, the two most prognostically contrasted tumor clusters in this cohort markedly differed in ROR2 expression (Fig. 6F). LMC class 1, composed of thin melanomas with a good prognosis and absence of proliferation markers [37], displays higher ROR2 levels than LMC class 3, composed of thicker melanomas with poor prognosis and a highly proliferative profile [37] (Fig. 6F). Altogether, these results indicate that by inhibiting cell proliferation ROR2 plays a protective role in primary melanomas since tumors expressing ROR2 remain thinner and have a better prognosis.

## Discussion

ROR2 acts either as a tumor suppressor or an oncogene depending on the tumor type [7], but, to our knowledge, it has never been shown to play both roles in the same tumor type. Previous investigations have established that ROR2 promotes cancer progression in melanoma by facilitating invasion and metastasis [27–29]. However, data presented in this article reveal that ROR2 has a dual role since it also inhibits melanoma proliferation and tumor growth. We have shown that by abolishing Akt phosphorylation, ROR2 regulates the expression of critical regulators of the cell-cycle. As a result, cell-cycle progression is delayed and cell proliferation is reduced. These observations are in agreement with both clinical and transcriptomic data from melanoma patients. Analysis of two datasets with more than 900 primary tumors revealed that *ROR2* levels have an inverse relationship with prognostic parameters associated with proliferation such as AJCC-stage, Breslow thickness, and mitotic rate. The tumor-suppressive functions of ROR2 are also supported by the observation that high *ROR2* levels correlated with a better prognosis of primary melanomas and is upregulated in patients showing transcriptomic signatures of good prognosis.

Although ROR2 has been involved in diverse processes in cancer, the implicated mechanisms have not been fully elucidated. It is worth noting that most of the reports to date describe that ROR2 has a positive effect on cell proliferation [12, 39, 40]. The mechanisms described implicate the activation of the Rho-family of small GTPases [39] or the upregulation of c-myc, Cyclin D1, Cyclin E, and CDK4 [40], and PCNA and CDK1 [12]. In contrast, other studies showed that ROR2 inhibited proliferation in gastric [26], colorectal [23], and ovarian cancer [41], as well as in esophageal squamous cell carcinoma [42]. However, these studies relied only on ectopic expression of ROR2 [26, 41, 42] or provided inconsistent results between overexpressing and silencing experiments [23]. Furthermore, the implicated mechanisms have not been investigated in detail [23, 26, 41, 42].

Our findings demonstrate that ROR2 inhibits proliferation by the inhibition of Akt activity and downstream signaling. The aberrant activation of the PI3K/Akt pathway has been consistently implicated in the development and/or clinical progression of melanoma. Among the critical processes positively regulated by Akt is cell proliferation. Akt signaling was shown to promote progression through the cell-cycle by regulating the levels and activity of diverse downstream factors involved in controlling the G1/S and G2/M transitions [43, 44]. Akt is a well known effector of the Wnt5a pathway and both Wnt5a and ROR1 were consistently shown to promote Akt phosphorylation in several tumor types, including melanoma [34]. In contrast, there is conflicting evidence regarding the effect of ROR2 on Akt. The PI3K/Akt pathway was shown to be activated by ROR2 and to be an effector of its oncogenic functions in osteosarcoma [45, 46], colon [47], breast cancer [48], and myeloma cells [49]. However, overexpression of ROR2 inhibited Akt phosphorylation in two breast cancer cell lines [42] and two pancreatic cancer cell lines [50]. By using both gain- and loss-of-function approaches we demonstrated that ROR2 strongly inhibits Akt phosphorylation in four melanoma cell lines. Interestingly, two of the cell lines in the present study (A375 and UACC903) were used to demonstrate that Wnt5a and ROR1 activate Akt [34] suggesting that both ROR receptors have antagonistic roles on the activation of Akt in melanoma. In addition, whereas ROR2 expression is a marker of good prognosis (this manuscript), ROR1 was shown to correlate with poor melanoma survival [34], further supporting the opposite function of both ROR1 and ROR2 in melanoma. This observation also provides a plausible explanation for the dual role of Wnt5a described in cancer [51] where it was shown to command a tumor-suppressing effect in colorectal cancer [52], neuroblastoma [53], ductal breast cancer [54], and leukemia [55] and an oncogenic role in melanoma, breast cancer, gastric cancer, pancreatic cancer, non-small-cell lung cancer, and prostate cancer [56, 57]. Future studies will determine whether a differential expression of ROR receptors in different tumor types is the reason for this dual behavior.

ROR2 has been proposed as a therapeutic target in cancer [5] and antibody-based therapeutic tools have been already developed [58, 59]. Further, the effect of targeting ROR2 is being evaluated in phase 1 and 2 clinical trials (NCT03504488, NCT03393936, and NCT03960060) for patients with advanced cancer. The present manuscript shows that ROR2 has a far more complex role than originally described and can even have both pro- and anti-tumorigenic roles in the same tumor type. Therefore, it promotes that the use of therapies against ROR2 to be preceded by studies intended to rule out anti-tumor effects of ROR2 in said tumor type.

## Conclusions

We have demonstrated that ROR2 inhibits Akt activity in melanoma and consequently it alters the expression, activity, and localization of major components of the cell-cycle regulatory machinery. As a result, ROR2 delays cell-cycle progression and reduces proliferation. These observations have important clinical implications since melanoma patients with high ROR2 levels results in thin primary tumors with a better prognosis.

## Supplementary Information

**Additional file 1:** List of antibodies used.

**Additional file 2: Figure S1. **(A) ROR2 inhibits cell growth. Crystal violet assays were performed in UACC903 cells stably transduced with either control (Empty) or a ROR2-expressing plasmid. Levels of ROR2 in these cells were determined by western blot. The analysis was performed as described for A375 in Fig. 1B. (B, C) Flow cytometry analysis of ROR2 levels in A375 and UACC903 stably transduced with either control (Empty) or a ROR2-expressing plasmid. The experiment was performed with either permeabilized (B) or intact (C) cells. Cells were collected and stained as described in “Methods”. Light-grey filled histogram corresponds to control (isotype) antibody and open histograms to ROR2 antibody. Bar graph shows the mean of ROR2 MFI (Mean Fluorescence Intensity) ± S.D. (from three independent experiments). Statistical significance was tested by a one-tailed Student’s T-Test, n = 3. The histograms displayed are representative of three independent experiments. (D) ROR2 levels upon overexpression are similar to those found in HeLa cells. ROR2 levels were assessed by western blot in HeLa, M2, A375-Empty, and A375-ROR2 cells. Bar graph shows the mean ± S.D. (from three independent experiments) of ROR2 levels normalized to the loading control. The blots displayed are representative of three independent experiments. Statistical significance was tested by ANOVA, n = 3. (E) ROR2 silencing increases cell growth. Crystal violet assays were performed in M2 cells upon silencing of ROR2. Levels of ROR2 in these cells were determined by western blot. The analysis was performed as described in Fig. 1B. (F) Efficient silencing of ROR2 in M2 and MeWo cells. Flow cytometry analysis of ROR2 levels in M2 and MeWo stably transduced with either control (scramble) plasmid or two shRNA for ROR2 (C2 and C4). Light-grey filled histogram corresponds to control (isotype) antibody and open histograms to ROR2 antibody. Bar graph shows the mean of ROR2 MFI (Mean Fluorescence Intensity) ± S.D. (from three independent experiments). Statistical significance was tested by a one-tailed Student’s T-Test (M2) or ANOVA (MeWo), n = 3. The histograms displayed are representative of three independent experiments. (G, H) ROR2 impairs cell-cycle progression. Flow cytometry analysis of PI-stained UACC903 cells overexpressing ROR2 (G) and M2 (H) cells upon silencing of ROR2. Bar graph shows the mean ± S.D. of the percentage of cells in each phase of the cell-cycle. The analysis was performed as described in Fig. 1E and F. *: p < 0.01, **: p < 0.001, ***: p < 0.0001, n = 3. n.s.: not significant. **Figure S2. **(A) LY294002 similarly inhibits p-Akt levels in both M2-scramble and M2-shROR2 cells. M2-scramble and M2-shROR2 cells were incubated with LY for the indicated times and protein extracts were analyzed by western blot with the indicated antibodies. GAPDH was used as loading control. Analysis was performed as described in Fig. 3A. (B) LY294002 decreases the viability of M2-shROR2 cells. M2-scramble and M2-shROR2 cells were incubated with the indicated concentrations of LY294002 for 48 h. Graphs show the mean ± S.D. of the percentage of viable cells. Statistical significance was tested by a one-tailed Student’s T-Test, n = 3. (C) Inhibition of Akt signaling does not induce apoptosis in cells with ROR2 knockdown. M2 and MeWo cells stably transduced with either control (scramble) plasmid or shRNA for ROR2 (C4 and C2) were incubated with 20 μM LY294002 for 48h. Cells were stained with Annexin V/Propidium Iodide and analyzed by flow cytometry. The dot plots displayed are representative of three independent experiments. The percent of cells in each quadrant is indicated. *: p < 0.01, **: p < 0.001, ***: p < 0.0001, n.s.: not significant.

## Data Availability

Data sharing is not applicable to this article as no datasets were generated during the current study.

## Abbreviations

FFPE: Formalin-fixed paraffin embedded
MSS: Melanoma-specific survival
LMC: Leeds melanoma cohort
LY: LY294002
