# Supplementary figures and images for: ROR2 has a protective role in melanoma by inhibiting Akt activity, cell-cycle progression, and proliferation
Source: J Biomed Sci. 2021 Nov 13;28:76. doi: 10.1186/s12929-021-00776-w (PMC8590781; doi:10.1186/s12929-021-00776-w)

**Figure S1**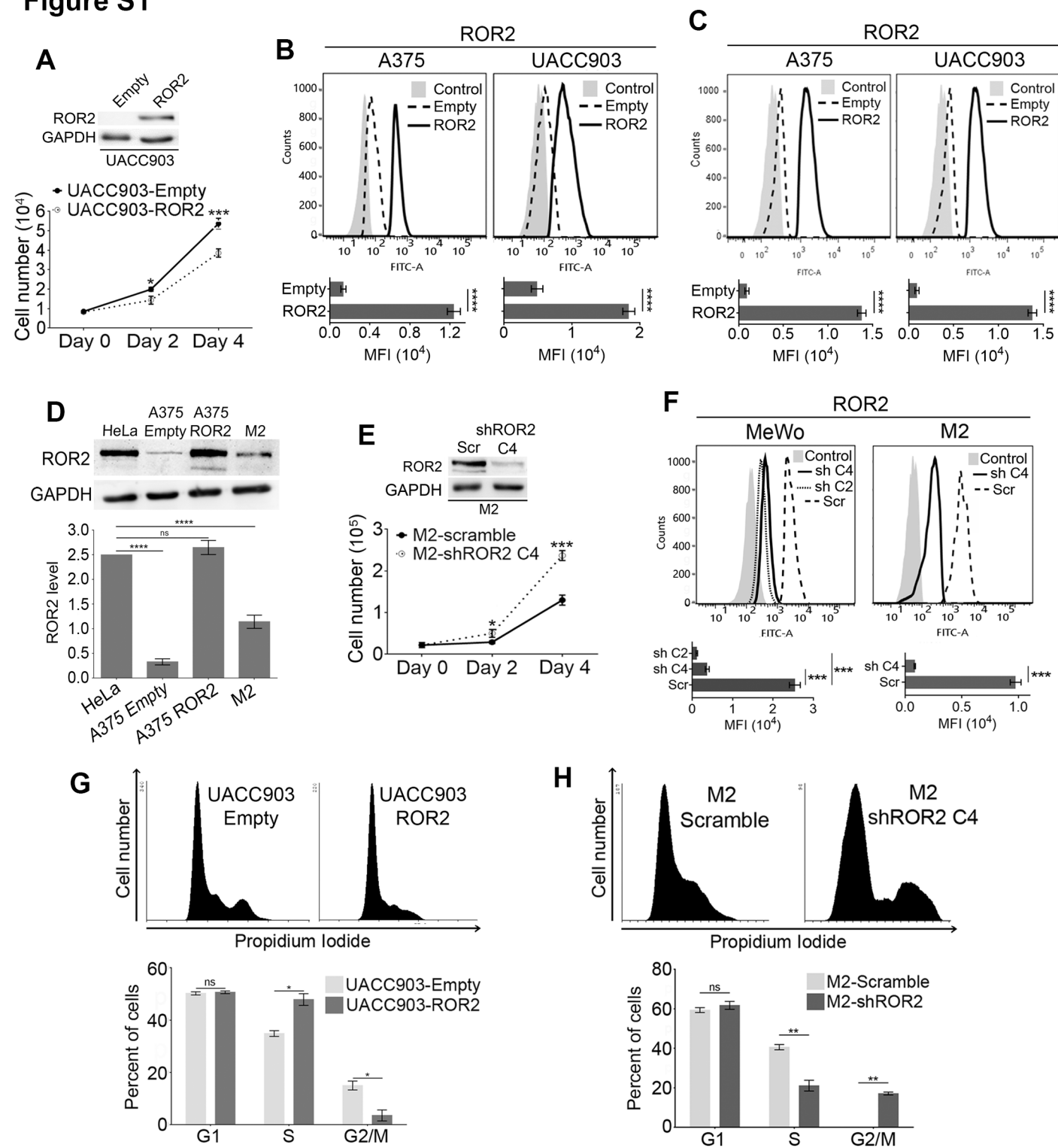

Figure S2

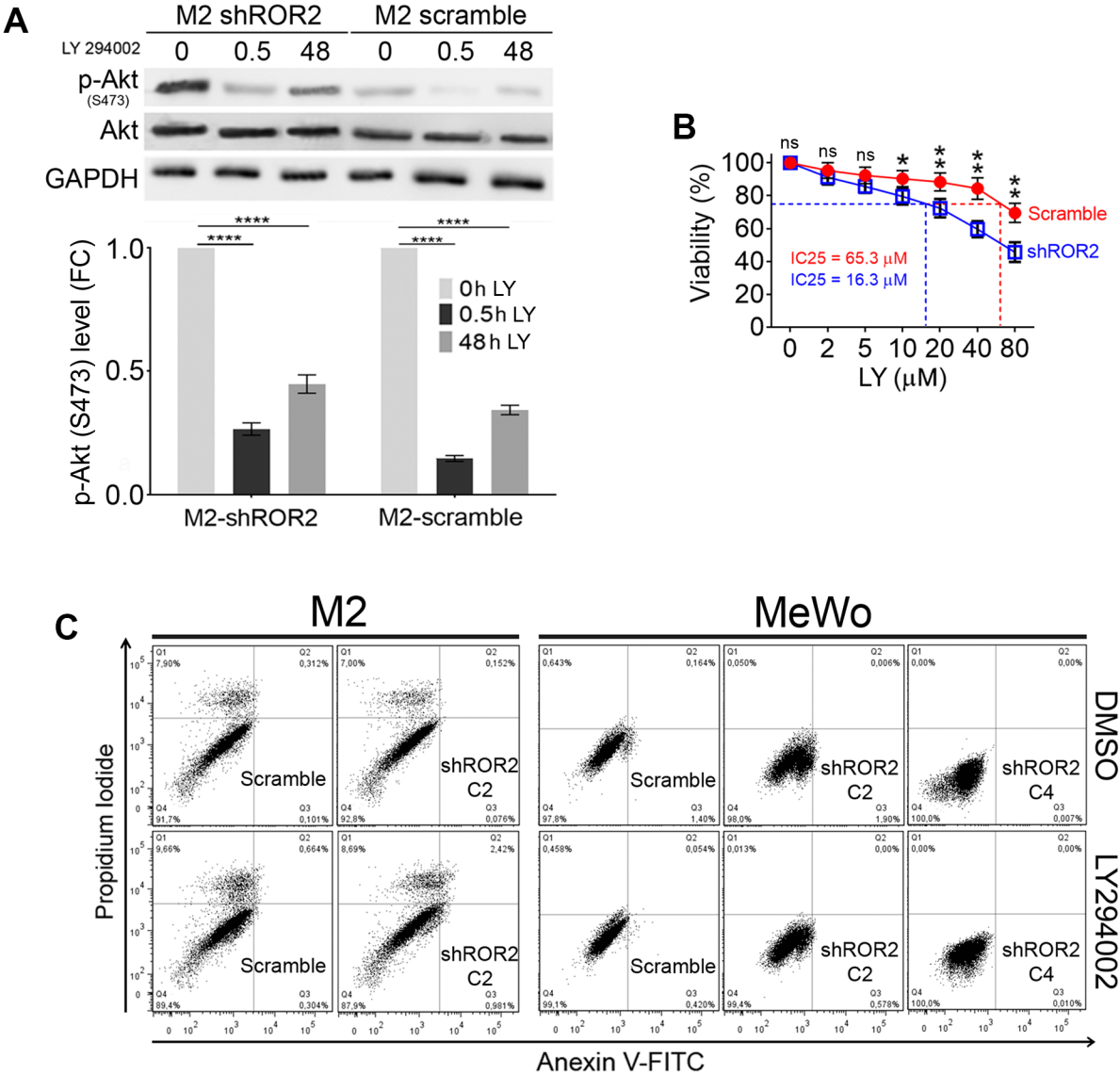

Supplement: Supplementary file 2 — Additional file 2: Figure S1. (A) ROR2 inhibits cell growth. Crystal violet assays were performed in UACC903 cells stably transduced with either control (Empty) or a ROR2-expressing plasmid. Levels of ROR2 in these cells were determined by western blot. The analysis was performed as described for A375 in Fig. 1B. (B, C) Flow cytometry analysis of ROR2 levels in A375 and UACC903 stably transduced with either control (Empty) or a ROR2-expressing plasmid. The experiment was performed with either permeabilized (B) or intact (C) cells. Cells were collected and stained as described in “Methods”. Light-grey filled histogram corresponds to control (isotype) antibody and open histograms to ROR2 antibody. Bar graph shows the mean of ROR2 MFI (Mean Fluorescence Intensity) ± S.D. (from three independent experiments). Statistical significance was tested by a one-tailed Student’s T-Test, n = 3. The histograms displayed are representative of three independent experiments. (D) ROR2 levels upon overexpression are similar to those found in HeLa cells. ROR2 levels were assessed by western blot in HeLa, M2, A375-Empty, and A375-ROR2 cells. Bar graph shows the mean ± S.D. (from three independent experiments) of ROR2 levels normalized to the loading control. The blots displayed are representative of three independent experiments. Statistical significance was tested by ANOVA, n = 3. (E) ROR2 silencing increases cell growth. Crystal violet assays were performed in M2 cells upon silencing of ROR2. Levels of ROR2 in these cells were determined by western blot. The analysis was performed as described in Fig. 1B. (F) Efficient silencing of ROR2 in M2 and MeWo cells. Flow cytometry analysis of ROR2 levels in M2 and MeWo stably transduced with either control (scramble) plasmid or two shRNA for ROR2 (C2 and C4). Light-grey filled histogram corresponds to control (isotype) antibody and open histograms to ROR2 antibody. Bar graph shows the mean of ROR2 MFI (Mean Fluorescence Intensity) ± S.D. (from [file 12929_2021_776_MOESM2_ESM.pdf]
